# Post-hospital mortality in children aged 2-12 years in Tanzania: A prospective cohort study

**DOI:** 10.1371/journal.pone.0202334

**Published:** 2018-08-14

**Authors:** Duncan K. Hau, Neema Chami, Aynsley Duncan, Luke R. Smart, Adolfine Hokororo, Neema M. Kayange, Robert N. Peck

**Affiliations:** 1 Department of Pediatrics, Weill Cornell Medical College, New York, New York, United States of America; 2 Department of Pediatrics, Catholic University of Health and Allied Sciences, Mwanza, Tanzania; 3 Department of Pediatrics, Bugando Medical Centre, Mwanza, Tanzania; 4 Center for Global Health, Weill Cornell Medical College, New York, New York, United States of America; 5 Department of Medicine, University of Washington, Seattle, Washington, United States of America; 6 Division of Hematology/Oncology, Department of Pediatrics, Cincinnati Children’s Hospital Medical Center, Cincinnati, Ohio, United States of America; Centers for Disease Control and Prevention, UNITED STATES

## Abstract

**Background:**

Sub-Saharan Africa has the highest rates of child mortality worldwide. Little is known about post-hospital outcomes after an index hospitalization for older children. We determined 12-month post-hospital mortality rate and identified factors associated with higher mortality.

**Methods:**

In this prospective cohort study, we enrolled children 2–12 years of age admitted to the pediatric wards of two public hospitals in northwestern Tanzania. Participants or proxies were contacted at 3, 6 and 12 months post-hospitalization. The primary outcome measured was mortality. Factors associated with mortality were determined using Cox regression analysis.

**Results:**

A total of 506 participants were enrolled. In-hospital mortality rate was 7.7% (39/506). Of the 467 participants discharged, the post-hospital mortality rate was 10.1% (47/467). Sickle cell disease (Hazard Ratio (HR) 3.32, 95% CI 1.44–7.68), severe malnutrition (HR 3.19, 95% CI 1.18–8.57), neurologic diseases (HR 3.51, 95% CI 1.35–9.11), heart disease (HR 7.11, 95% CI, 2.89–17.51), cancer (HR 11.79, 95% CI 4.95–28.03), and septic shock (HR 4.64, 95% CI 1.42–15.08) had higher association with mortality compared to other diagnoses. The risk factors significantly associated with mortality included older age (HR 1.01, 95% CI 1.00–1.08), lower hemoglobin level (HR 0.83, 95% CI 0.76–0.90), lower Glasgow Coma Scale (HR 0.66, 95% CI 0.59–0.74), history of decreased urine output (HR 2.87, 95% CI 1.49–5.53), higher respiratory rate (HR 1.02, 95% CI 1.00–1.03), estimated glomerular filtration rate less than 60 ml/min/1.73m^2^ (binary) (HR 1.84, 95% CI 1.10–3.10), and lower oxygen saturation (HR 0.96, 95% CI 0.92–0.99).

**Conclusions:**

Post-hospital mortality is disturbingly high among children 2–12 years of age in Tanzania. Post-hospital interventions are urgently needed especially for older children with chronic illnesses.

## Introduction

Despite dramatic improvements in childhood mortality in recent decades, around 16,000 children continue to die every day around the world, with the highest rates of child mortality in Africa [1]. Most efforts to reduce child mortality in Africa have focused on either prevention of disease or treatment of acute illnesses, and little attention has been given to understanding what happens to children after they leave the hospital [2–3]. In particular, little is known about post-hospital outcomes for African children [4].

Infectious diseases, such as diarrhea and pneumonia, are the leading causes of childhood mortality, with the highest proportion of death occurring between birth to 2 years [5]. The few available reports of post-hospital outcomes for children in sub-Saharan Africa have focused mainly on young children with a specific diagnosis, like pneumonia or diarrhea [4]. Data are lacking regarding post-hospital outcomes for children above 2 years of age with a broad range of diagnoses.

Therefore, we conducted a prospective cohort study of children aged 2–12 years hospitalized in the pediatric wards of two public hospitals in northwestern Tanzania. Our study objectives were: 1) to determine the mortality rate for children aged 2–12 years during the twelve months after an index hospitalization, 2) to determine when most deaths occurred (in-hospital vs. post-hospital), and 3) to identify factors associated with higher mortality including any common diseases.

## Methods

### Study site

In this prospective cohort study, we consecutively screened and enrolled children hospitalized on the pediatric wards of 2 public hospitals. Bugando Medical Center (BMC) is a tertiary hospital and serves as the zonal referral hospital for northwestern Tanzania with a catchment area of approximately 13 million people. BMC has 1,000 inpatient beds and 3,500 pediatric hospitalizations per year. Sekou-Toure Hospital (STH) is a regional referral hospital for Mwanza Region with a population of approximately 3 million people. STH has 320 inpatient beds and 2,000 pediatric hospitalizations per year. Both BMC and STH are located in the city of Mwanza, the second largest city in Tanzania and the capital of the Mwanza region.

### Study population and inclusion criteria

All children between 2–12 years of age hospitalized in the BMC or STH pediatric wards were eligible for enrollment in the study. Patients above 12 years were excluded since they are admitted to the adult wards at BMC and STH. The parent or guardian of a potential participant was provided with information by a hospital staff regarding the study within 12 hours of hospitalization. Children were enrolled only after obtaining informed consent from a parent or guardian by a study member. Study participants with multiple hospitalizations during the study period were only enrolled during their first hospitalization.

### Study procedure

On the day of enrollment, a modified version of the WHO STEPS questionnaire was administered in Kiswahili by a study investigator to the parent or guardian [6]. The study investigator was independent from the clinical team caring for the patient in the hospital. The WHO STEPS questionnaire includes questions regarding home setting, medical history, prior testing, diagnosis and treatment for diseases as well as standard protocols for physical examination. After completing the questionnaire, the study investigator conducted a standardized physical examination including the measurement of vital signs, weight and height. Temperature was taken axillary. Weight was measured to the nearest 0.1 kg using a digital scale (DETECTO, USA), which was adjusted to zero before each measurement. For participants unable to stand, their weight was taken on a hanging scale. Height was measured to the nearest 0.1 cm using a stadiometer. For participants unable to stand, their height was measured while lying down.

### Laboratory analysis

At the time of hospitalization, by national policy, all children were offered testing for Human Immunodeficiency Virus (HIV) according to the Tanzania national guidelines for provider-initiated testing and counseling (PITC) [7]. All participants also underwent measurements of glucose, creatinine, hemoglobin, and urine dipstick testing as standard procedures of hospitalization at BMC and STH. Serum creatinine levels were measured using a Cobas Integra 400 Plus Analyzer (Roche Diagnostic Limited, Switzerland). An estimated glomerular filtration rate (eGFR) was calculated using the bedside Schwartz equation as recommended by international guidelines [8]. Random blood glucose was measured using a finger stick sample (Ascensia Glucometer, Bayer Healthcare, Germany). A urine dipstick was used to test for proteinuria and hematuria (Multistix 10SG, Siemens, USA).

### Discharge diagnoses

Diagnoses were determined at the time of hospital death or discharge. Since 2013, BMC and STH have used a standard list of recommended pediatric discharge diagnoses. These diagnoses were adapted from the WHO’s International Classification of Diseases version 10 (ICD-10) [9]. For a child with dual or multiple diagnoses (ie: severe malnutrition and diarrheal diseases), a single diagnosis was recorded for this study that was based on the primary diagnosis recorded by the clinicians caring for the child.

### Follow-up of participants

Three mobile phone numbers were obtained from all participants’ caretakers at the time of discharge: one number for the study participant’s parent or guardian and two additional numbers for relatives or close friends. Caretakers were given standard discharge instructions and told to follow-up in clinic within 2 weeks of discharge or sooner if necessary. Follow-up phone calls were made at 3, 6 and 12 months post-discharge. During each call, a standard set of questions was asked in Kiswahili including 1) vital status of the participant and 2) clinic attendance. If the participant had died, the date of death was also determined.

### Measures

The primary study outcome was mortality. Mortality was classified as in-hospital if it occurred during the index hospitalization and post-hospital if it occurred in the 12 months that followed the index hospitalization.

### Data analysis

Data were entered into Microsoft Excel (Microsoft, Redmond, Washington, USA) and analyzed using STATA version 14 (College Station, Texas, USA). Non-missing data were included in all calculations. No variable was missing for more than 14 participants. Categorical variables were described as proportions (percentages), and continuous variables were described as means (standard deviations). Univariate and multivariate Cox regression analysis was used to determine factors associated with mortality. All factors significantly associated with mortality in the univariate analysis were included in the multivariate analysis except for diagnosis category. Hazard ratios (HR) and their 95% confidence intervals (CI) were reported with p-value of less than 0.05 regarded as statistically significant.

Kaplan-Meier survival curves were used to display incident mortality. Study participants lost to follow-up were censored at the last contact date. A log-rank test was used to determine if mortality incidence differed by diagnosis category.

### Ethical consideration

Permission to conduct the study was obtained from the research committees of Bugando Medical Center, Sekou-Toure Hospital, Weill Cornell Medical College, and the National Institute for Medical Research in Tanzania. Participants were enrolled only after obtaining informed consent from one of their parents or guardian. Parents also consented to receive phone calls at either their own mobile phone number or the mobile phone numbers they provided for relatives or friends. They agreed that if they were not available to receive the phone call, relatives or friends could provide information about the vital status of the participant. Disease management was conducted by the clinicians in accordance with the hospital and Tanzanian management protocols.

## Results

### Study enrollment

From 1^st^ August 2014 to 30^th^ November 2014, 537 children between the ages of 2–12 years were hospitalized in the pediatric wards of BMC and STH. Of the 537 children hospitalized, 15 died before enrollment occurred, 8 were excluded for being referral cases from another hospital, and 8 declined participation. The remaining 506 children (94.2%) were enrolled, with 461/506 (91.1%) at BMC and 45/506 (8.9%) at STH. In-hospital mortality occurred for 39 participants (7.7%). Of the 467 participants who were discharged, mobile phone contact was made with 458/467 (98.1%) participants’ parents or designated proxies at 3 months, 409/467 (87.6%) at 6 months, and 372/467 (79.7%) at 12 months.

### Baseline characteristics

Among the participants, 214/506 (42.3%) were female (Table 1). The mean age was 54.5 months ± 32.5 standard deviation (SD), with 176/506 (34.8%) being 5 years of age or older. The most common reported symptom on history was fever (72.9%), however only 26.9% of participants were found to have a temperature ≥ 38.0° Celsius upon admission. HIV-infected participants were 5.9%. On physical examination, 14.0% of participants had severe malnutrition based on anthropometric measurements. On laboratory investigations, 20.9% had an eGFR of < 60 ml/min/1.73m^2^, 19.2% had proteinuria and the mean hemoglobin level was 7.9 g/dL (± 2.7).

**Table 1:** Baseline characteristics of participants at time of admission.

Variable	Study Participants (N = 506)
Demographic Characteristics	
Female	214 (42.3)
Age, months, mean (SD)	54.5 (32.5)
Under 5 years	330 (65.2)
5–12 years	176 (34.8)
Pit latrine at home	304 (60.1)
Lake or pond as water source	192 (37.4)
HIV Status	
Negative	475 (93.9)
Positive	30 (5.9)
Refused testing	1 (0.2)
Symptoms Reported on Hospitalization	
Fever	369 (72.9)
Diarrhea	143 (28.2)
Vomiting	138 (27.3)
Decreased urine output	30 (5.9)
Signs on Physical Examination	
Temperature, Celsius, mean (SD)	37.3 (1.0)
Temp ≥ 38.0	136 (26.9)
Heart rate, beats per minute, mean (SD)	
2–5 years	117.6 (22.1)
6–12 years	107.7 (20.4)
Systolic blood pressure, mm Hg, mean (SD)	
2–5 years	90.4 (12.9)
6–12 years	102.9 (13.9)
Diastolic blood pressure, mm Hg, mean (SD)	
2–5 years	60.0 (9.5)
6–12 years	67.0 (10.4)
Respiratory Rate, breaths per minute, mean (SD)	
2–5 years	34.2 (12.7)
6–12 years	28.6 (11.4)
Oxygen saturation, percentage, mean (SD)	95.7 (4.9)
Nutritional status	
Normal	264 (52.2)
Mild Malnutrition *	95 (18.8)
Moderate Malnutrition +	76 (15.0)
Severe Malnutrition Δ	71 (14.0)
Glascow Coma Score (GCS)	
< 13	16 (3.2)
13–14	11 (2.2)
15	479 (94.7)
Bilateral lower extremity edema	67 (13.2)
Laboratory Investigation on Hospitalization	
Random blood glucose, mg/dL, mean (SD)	106 (88.3)
Estimated glomerular filtration rate (eGFR), ml/min/1.73m^2^, mean (SD)	113.5 (59.9)
< 30	18 (3.6)
30–59	88 (17.4)
≥ 60	400 (79.1)
Proteinuria by urinalysis	97 (19.2)
Hematuria by urinalysis	20 (4.0)
Hemoglobin level, g/dL, mean (SD)	7.9 (2.7)
Diagnosis Category	
Malaria	64 (12.7)
Sickle cell disease	61 (12.1)
Diarrheal diseases	60 (11.9)
Respiratory infections	60 (11.9)
Anemia	49 (9.7)
Urinary tract infection	36 (7.1)
Severe malnutrition	35 (6.9)
Neurologic diseases	33 (6.5)
Heart disease	23 (4.5)
Cancer	20 (3.9)
Septic shock	14 (2.7)
Other	50 (9.8)

Data are presented as number (percentage) of study participants unless otherwise indicated.

* Weight-for-Height Z score < -1 and ≥ -2 SD

+ Weight-for-Height Z score < -2 and ≥ -3 SD

Δ Weight-for-Height Z score < -3 SD

The top four diagnosis categories of the 506 study participants were as follows: 64 (12.7%) malaria, 61 (12.1%) sickle cell disease, 60 (11.9%) diarrheal diseases, and 60 (11.9%) respiratory infections.

### Outcomes

**Fig 1:**
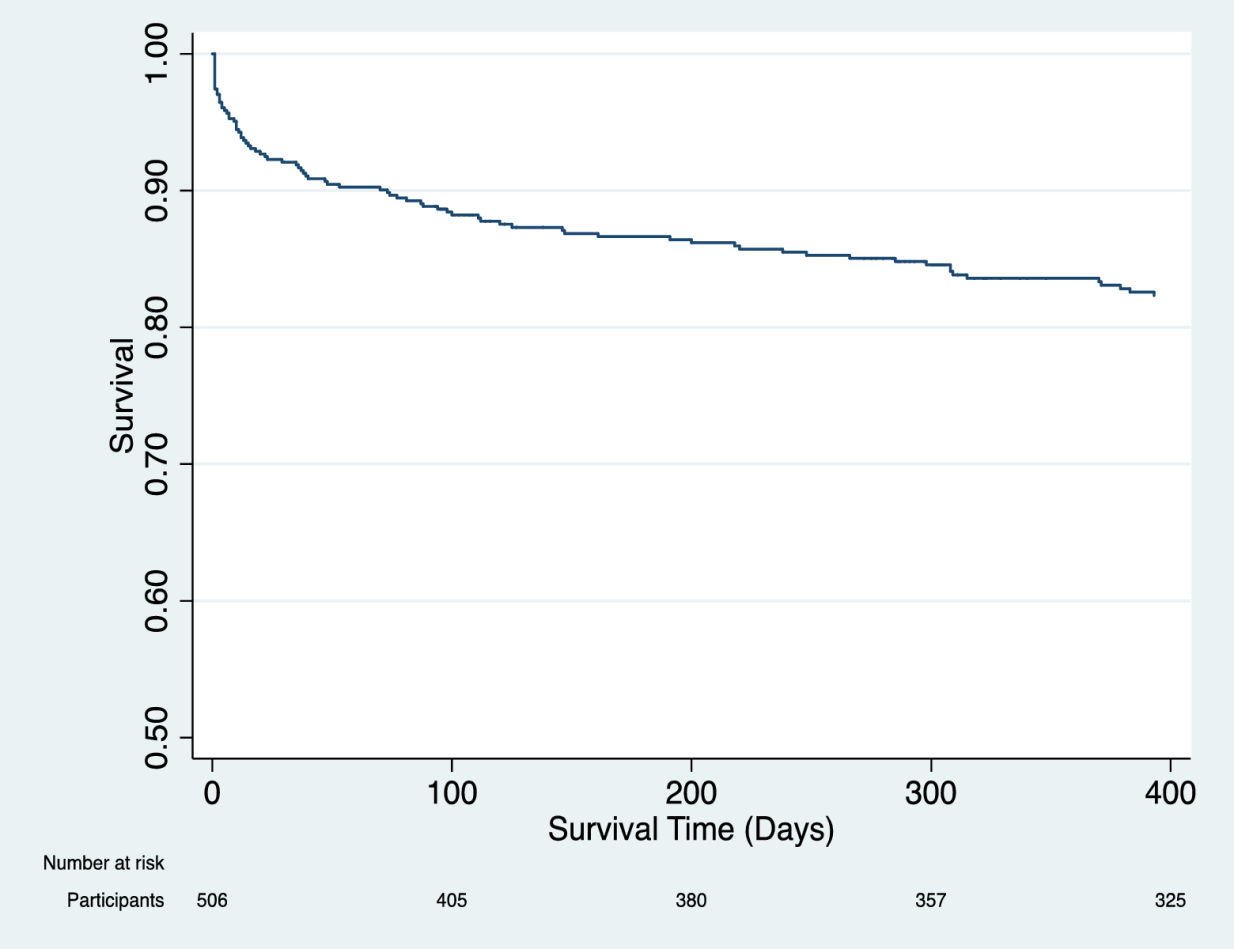
Survival curve for all participants.

Of the 506 participants hospitalized, 86 (17.0%) died within 12 months of admission. Excluding the 95 participants lost to follow-up, the overall mortality rate was 20.9% (86/411). Of the 86 deaths, 47 (54.7%) occurred post-hospital. The post-hospital mortality rate was 10.1% (47/467). Excluding the 95 participants lost to follow-up, the post-hospital mortality rate was 12.6% (47/372). The median time-point for death was 37 days after admission for all participants (Fig 1). For post-hospital mortality, the median time-point for death was 120 days after discharge (S1 Fig). The average length of hospitalization was 8 days.

Of the 467 participants discharged, 210 (45.0%) participants’ families reported no follow-up in clinic at 3 months after discharge. Of the 47 participants who died post-discharge, 25 (53.2%) participants’ families reported no follow-up in clinic at 3 months after discharge. For these 25 participants, 52.0% (13/25) of deaths occurred before the 3 month time point. The median time-point for death for participants with no follow-up was 80 days after discharge.

### Factors associated with mortality

In our univariate analysis, we tested all the variables in Table 1 to determine which were significantly associated with overall mortality. Table 2 contains those factors which were significantly associated (p<0.05) with overall motility. All other factors not listed were not significantly associated. For the full univariate analysis, see S1 Table.

**Table 2:** Factors significantly associated (p<0.05) with overall mortality by univariate analysis.

Variable	Total N = 506	Dead N = 86	Alive N = 420	Hazard Ratio (95% CI)	p-value
Demographic Characteristics					
Age, months, mean (SD)	506	64.0 (36.5)	52.6 (31.3)	1.01 (1.00–1.01)	0.004
Categorical Age					
Under 5 years	330	45 (13.6)	285 (86.4)	Ref	
5–12 years	176	41 (23.3)	135 (76.7)	1.75 (1.15–2.68)	0.009
Pit latrine at home					
Yes	304	59 (19.4)	245 (80.6)	1.58 (1.00–2.50)	0.04
No	202	27 (13.4)	175 (86.6)	Ref	
Symptoms Reported on Hospitalization					
Decreased urine output					
Yes	30	15 (50.0)	15 (50.0)	4.95 (2.83–8.66)	<0.001
No	476	71 (14.9)	405 (85.1)	Ref	
Signs on Physical Examination					
Oxygen saturation, percentage, mean (SD)	506	93.2 (7.7)	96.2 (3.9)	0.93 (0.91–0.95)	<0.001
GCS (ordinal)					
< 13	16	11 (68.8)	5 (31.2)	0.66 (0.60–0.73)	<0.001
13–14	11	6 (54.5)	5 (45.5)		
15	479	69 (14.4)	410 (85.6)		
Bilateral lower extremity edema					
Yes	67	20 (29.9)	47 (70.1)	2.31 (1.40–3.81)	0.001
No	439	66 (15.0)	373 (85.0)	Ref	
Respiratory Rate, breaths per minute, mean (SD)					
2–5 years	362	41.2 (16.9)	33.0 (11.5)	1.04 (1.02–1.06)	<0.001
6–12 years	144	32.1 (9.4)	27.5 (11.8)	1.02 (1.00–1.04)	0.03
Diastolic blood pressure, mm Hg, mean (SD)					
6–12 years	144	63.2 (13.4)	68.1 (9.0)	0.94 (0.91–0.98)	0.005
Laboratory Investigation on Hospitalization					
Hemoglobin level, g/dL, mean (SD)	505	6.5 (2.8)	8.1 (2.6)	0.82 (0.75–0.88)	<0.001
Proteinuria by urinalysis (binary)					
Positive	97	28 (28.9)	69 (71.1)	2.38 (1.51–3.74)	<0.001
Negative	409	58 (14.2)	351 (85.8)	Ref	
Hematuria by urinalysis (binary)					
Positive	20	8 (40.0)	12 (60.0)	2.81 (1.35–5.81)	0.005
Negative	486	78 (16.0)	408 (84.0)	Ref	
eGFR < 60 ml/min/1.73m^2^ (binary)					
Yes	106	27 (25.5)	79 (74.5)	1.91 (1.21–3.02)	0.005
No	400	59 (14.8)	341 (85.2)	Ref	
Diagnosis Category					
Cancer	20	12 (60.0)	8 (40.0)	11.79 (4.95–28.03)	<0.001
Heart disease	23	10 (43.5)	13 (56.5)	7.11 (2.89–17.51)	<0.001
Sickle cell disease	61	14 (23.0)	47 (77.0)	3.32 (1.44–7.68)	0.005
Neurologic diseases	33	8 (24.2)	25 (75.8)	3.51 (1.35–9.11)	0.01
Septic shock	14	4 (28.6)	10 (71.4)	4.64 (1.42–15.08)	0.01
Severe malnutrition	35	7 (20.0)	28 (80.0)	3.19 (1.18–8.57)	0.02

Data are presented as number (percentage) of study participants unless otherwise indicated.

Table 3 lists the multivariate analysis for factors associated with overall mortality. The factors that remained significantly associated with mortality included older age (HR 1.01 [95% CI 1.00–1.08], p<0.001), history of decreased urine output (HR 2.87 [95% CI 1.49–5.53], p = 0.002), higher respiratory rate (HR 1.02 [95% CI 1.00–1.03], p = 0.010), and eGFR less than 60 ml/min/1.73m^2^ (binary) (HR 1.84 [95% CI 1.10–3.10], p = .02). The factors that were protective include hemoglobin level (HR 0.83 [95% CI 0.76–0.90], p<0.001), GCS (HR 0.66 [95% CI 0.59–0.74], p<0.001), and oxygen saturation (HR 0.96 [95% CI 0.92–0.99], p = 0.04). The univariate and multivariate analysis for factors associated with mortality post-discharge are listed in S2 Table and S3 Table.

**Table 3:** Factors significantly associated (p<0.05) with overall mortality by multivariate analysis.

Variable	Hazard Ratio (95% CI)	p-value
Age	1.01 (1.00–1.08)	<0.001^¶^
Hemoglobin level	0.83 (0.76–0.90)	<0.001^¶^
GCS (ordinal: <13, 13–14, 15)	0.66 (0.59–0.74)	<0.001^¶^
History of decreased urine output	2.87 (1.49–5.53)	0.002^¶^
Respiratory rate	1.02 (1.00–1.03)	0.01^¶^
eGFR (binary: < 60 ml/min/1.73m^2^)	1.84 (1.10–3.10)	0.02^¶^
Oxygen saturation	0.96 (0.92–0.99)	0.04^¶^
Pit latrine at home	1.61 (0.98–2.65)	0.05
Bilateral lower extremity edema	1.44 (0.82–2.53)	0.20
Proteinuria by urinalysis	1.28 (0.75–2.19)	0.36
Hematuria by urinalysis	1.37 (0.60–3.08)	0.44

¶ P value significant (<0.05)

The mortality rate in children 9–12 years old was 30.2% (19/63), versus 17.3% (14/81) in children 6–8 years old and 14.6% (53/362) in children 2–5 years old. The mortality rate in children with an admission hemoglobin level ≤ 5 g/dL was 30.5% (39/128), versus 12.5% (47/377) for children with a hemoglobin level > 5 g/dL.

**Fig 2:**
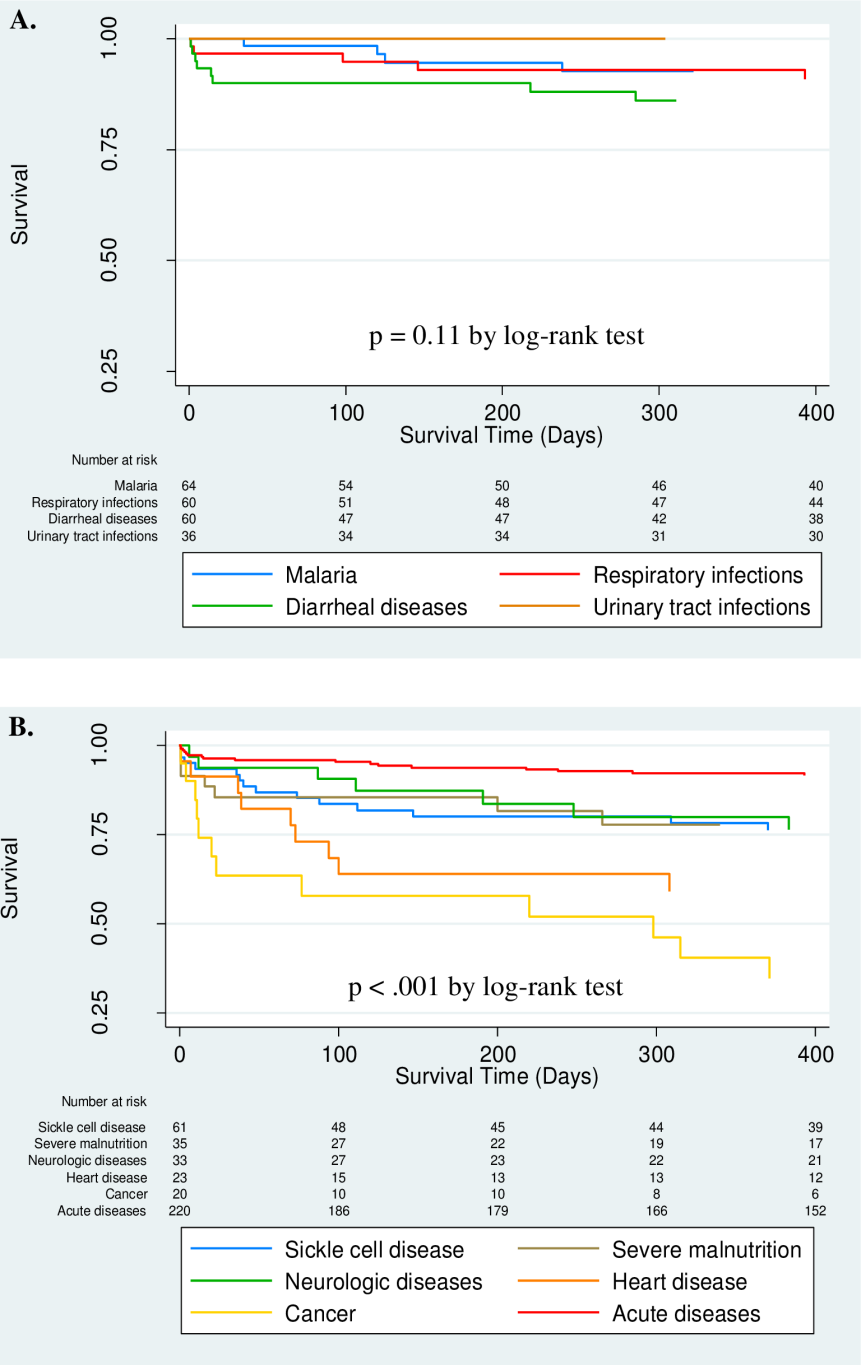
Survival curves by diagnostic categories among acute and chronic diseases. (A) Acute diseases; (B) Chronic diseases and acute diseases combined from Fig 2A.

When comparing mortality among acute diseases (malaria, respiratory infections, diarrheal diseases and urinary tract infections), mortality did not vary significant (p = 0.11 by log-rank test) (Fig 2A). However, mortality did vary significantly (p<0.001 by log-rank test) between chronic diseases (sickle cell disease, severe malnutrition, neurologic diseases, heart disease and cancer) and acute diseases (Fig 2B).

## Discussion

The mortality rates of hospitalized children in Africa both during their acute admission and in the months that follow discharge are striking. In our study, nearly 20% of children aged 2–12 years old admitted to BMC and STH died within 12-months of hospitalization, and 55% of those deaths occurred during the post-hospital period. This confirms and extends findings from prior studies conducted in Kenya and Malawi where post-hospital mortality was higher than in-hospital mortality [10–11]. Zucker et al. reported an in-hospital mortality of 10% compared to a post-hospital mortality of 13% over an eight-week follow-up period for children 5 years of age and under regardless of their diagnosis. Phiri et al. reported an in-hospital mortality of 6% compared to a post-hospital mortality of 12% over an eighteen-month follow-up period for children 5 years or age and under with severe anemia. We demonstrate that even children up to 12 years of age admitted in the general pediatric wards with a broad range of diagnoses suffer from alarming rates of post-hospital mortality.

To put in context, the 2016 Tanzania country estimates of mortality rate for under-five were 60 deaths per 1,000 live male births and 53 deaths per 1,000 live female births. For children aged 5–14, the probability of dying was estimated to be 12 deaths per 1,000 children aged 5 [12]. In our study for children under-five there were 21 post-hospital deaths for 306 children under-five discharged (S2 Table), which is equivalent to 68 deaths per 1,000 live births. In our study for children aged 5–12, there were 26 post-hospital deaths for 161 children discharged, which is equivalent to 161 deaths per 1,000 lives. A greater understanding of access and barriers to post-hospital care for children of all ages in Africa are needed to reduce childhood mortality.

Chronic diseases such as sickle cell disease, severe malnutrition, neurologic diseases, heart disease, and cancer had significantly higher post-hospital mortality than acute diseases (S1 Table). Sickle cell disease along account for 21% (10/47) of the deaths in the first year after hospital discharge. Our study also found older age to be a significant factor associated with mortality. Based on our experience of working in a resource-poor country, possible explanations for chronic illnesses and older age being factors associated with mortality include the following. Older children are more likely to have chronic diseases and have experienced multiple sequelae after living with their chronic diseases for several years, leaving them more susceptible to a new insult. Furthermore, in resource poor countries, older children often do not reach medical attention until their diseases have reached an advanced stage. This may be a result of parents delaying medical care because they are unable to afford time or money for travel, are reluctant for a child to miss school or simply hope that an illness will be self-resolving. Other times parents may seek treatment from traditional medicine healers before bringing the child to a hospital [13]. Although acute infectious diseases like respiratory and diarrheal infections are the leading causes of death in young children [5], chronic illnesses appear to have significantly higher mortality for older children compared to acute illnesses. Further research and interventions to reduce child mortality should focus not only on young children with acute infectious diseases, but also on older children with chronic illnesses.

A high mortality rate in the post-discharge period, as our study and others have shown [10–11, 14–16], supports the idea of a tenuous transition from hospital to home. High post-hospital mortality may be explained partly by the “post-hospital syndrome”, which has been described as an acquired transient period of vulnerability following discharge [17]. Stressors experienced during hospitalization like sleep deprivation, poor nutrition, pain, and adverse medication effects can contribute to a vulnerable state where a patient is more prone to deterioration after leaving the hospital. Another factor likely contributing to high mortality in the post-discharge period is poor linkage from hospitals to primary care clinics. Studies from the US have shown where patients who are discharged from hospitals and have early linkage to follow-up primary care providers have better outcomes [18–19]. Early and intensive follow-up for children discharged from hospitals is crucial. However, in a resource poor country like Tanzania, we speculate linkage to primary care clinic is poor and a contributor to post-hospital mortality.

Improving the critical transition from hospital to home should be a high priority for pediatric wards in Africa. Cost-effective, feasible and novel interventions are needed for where the burden of child mortality is greatest. A post-hospital intervention study conducted in Uganda, which included post-discharge referrals for follow-up visits and a discharge kit that had brief educational counseling along with preventive items (soap, a mosquito net, and oral rehydration salts) given at the time of discharge, was shown to affect care during illness recovery and lead to improved outcomes for children under 5 years of age [20]. Another promising possibility of post-hospital intervention that has been successful with a chronic illness like HIV in the adult population, is the use of case management intervention designed to link HIV-infected patients to primary care [21–22]. We are currently developing a post-hospitalization case management program focused on children with chronic illnesses at BMC to improve post-hospital outcomes.

Our study has limitations. These results come from 2 hospitals in Africa, although BMC and STH are similar to other public hospitals in East Africa [23–24]. Another limitation is that 20% of children were lost to follow-up by 12 months. On the other hand, follow-up rates at 3 and 6 months were 90%. The 20% of children who were lost-to-follow-up in this study are possibly more likely to have died than the subjects whose parents were available for phone calls. This could make our striking findings an underestimate of the true burden of post-hospital mortality.

In conclusion, we conducted a prospective cohort study with 506 children aged 2–12 years of age hospitalized on the general pediatric wards of 2 public hospitals in Tanzania and followed for one-year post-discharge. Children have nearly a 20% chance of mortality following admission over a 12-month period, with over 50% of mortality occurring post-hospital. Novel and sustainable interventions during the post-discharge period targeting a subset of children, particularly older children with chronic illnesses, are urgently needed to reduce overall childhood mortality in Africa.

## Supporting information

S1 Fig Survival curve for participants discharged from hospital. (TIF)

S1 Table Univariate Cox regression analysis for factors associated with overall mortality. (DOCX)

S2 Table Univariate Cox regression analysis for factors associated with post-hospital mortality. (DOCX)

S3 Table Multivariate Cox regression analysis for factors associated with post-hospital mortality. (DOCX)
