# Supplementary figures and images for: Post-hospital mortality in children aged 2-12 years in Tanzania: A prospective cohort study
Source: PLoS One. 2018 Aug 14;13(8):e0202334. doi: 10.1371/journal.pone.0202334 (PMC6091952; doi:10.1371/journal.pone.0202334)

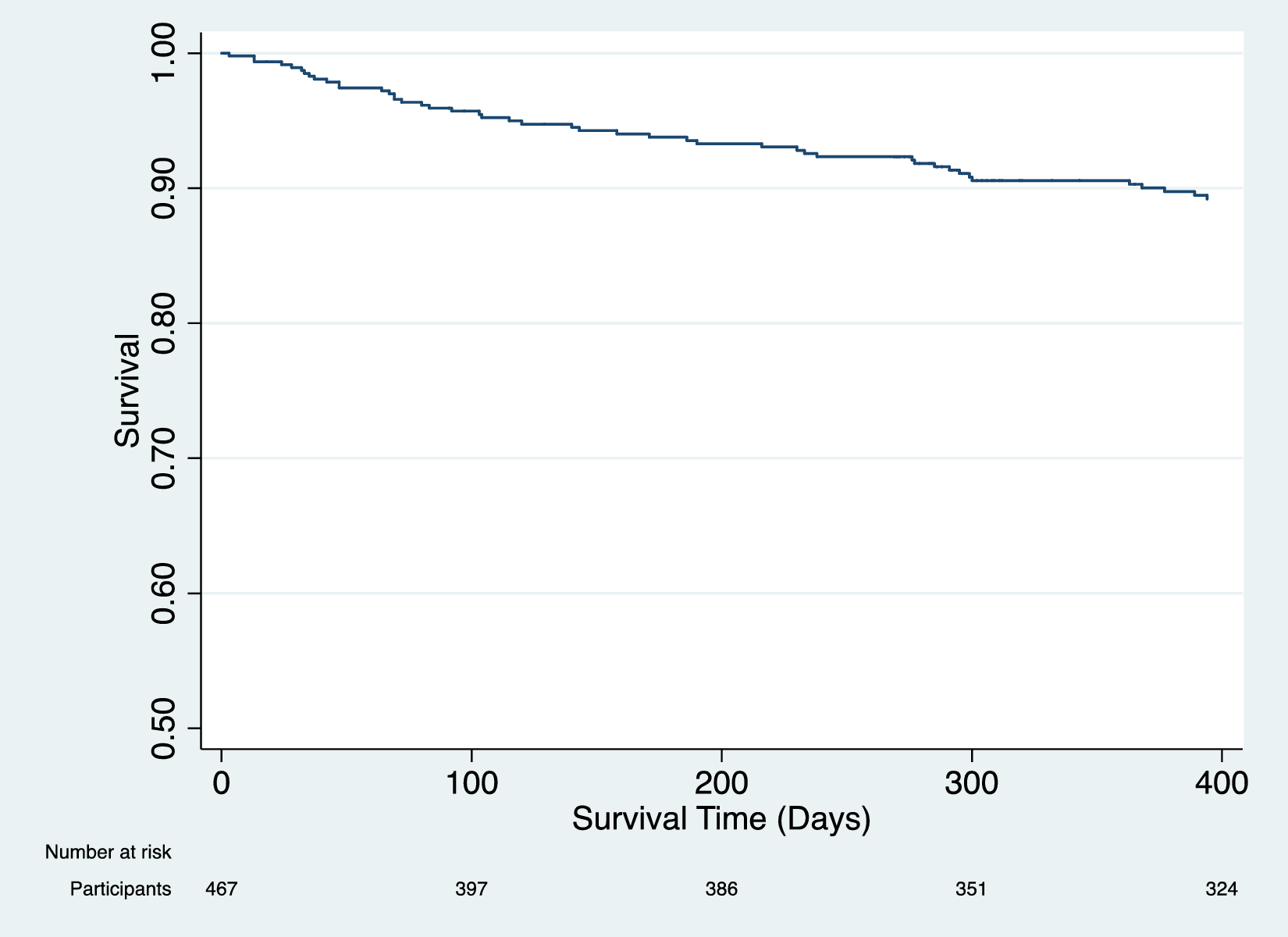

Supplement: S1 Fig — (TIF) [file pone.0202334.s001.tif]
