# Supplementary material for: Post-hospital mortality in children aged 2-12 years in Tanzania: A prospective cohort study
Source: PLoS One. 2018 Aug 14;13(8):e0202334. doi: 10.1371/journal.pone.0202334 (PMC6091952; doi:10.1371/journal.pone.0202334)
Supplement: S1 Table — (DOCX) [file pone.0202334.s002.docx]

**S1 Table. Univariate Cox regression analysis for factors associated with overall mortality.**

| **Variable** | **Total**  **N=506** | **Dead**  **N=86** | **Alive**  **N=420** | **Hazard Ratio**  **(95% CI)** | | **p-value** |
| --- | --- | --- | --- | --- | --- | --- |
| Demographic Characteristics |  |  |  |  | |  |
| Age, months, mean (SD) | 506 | 64.0 (36.5) | 52.6 (31.3) | 1.01 (1.00 – 1.01) | | 0.004^¶^ |
| Categorical Age | | | | | | |
| Under 5 years | 330 | 45 (13.6) | 285 (86.4) | Ref | |  |
| 5 – 12 years | 176 | 41 (23.3) | 135 (76.7) | 1.75 (1.15 – 2.68) | | 0.009^¶^ |
| Pit latrine at home | | | | | | |
| Yes | 304 | 59 (19.4) | 245 (80.6) | 1.58 (1.00 – 2.50) | | 0.04^¶^ |
| No | 202 | 27 (13.4) | 175 (86.6) | Ref | |  |
| Sex | | | | | | |
| Male | 292 | 46 (15.8) | 246 (84.2) | 0.84 (0.55 – 1.28) | | 0.42 |
| Female | 214 | 40 (18.7) | 174 (81.3) | Ref | |  |
| Lake or pond as water source | | | | | | |
| Yes | 192 | 34 (17.7) | 158 (82.3) | 1.10 (0.71 – 1.69) | | 0.67 |
| No | 314 | 52 (16.6) | 262 (83.4) | Ref | |  |
| HIV Status |  |  |  |  | |  |
| Positive | 30 | 6 (20) | 24 (80) | 1.38 (0.60 – 3.16) | | 0.44 |
| Negative | 476 | 80 (16.8) | 396 (83.2) | Ref | |  |
| Symptoms Reported on Hospitalization | |  |  |  | |  |
| Decreased urine output |  |  |  |  | |  |
| Yes | 30 | 15 (50.0) | 15 (50.0) | 4.95 (2.83 – 8.66) | | <0.001^¶^ |
| No | 476 | 71 (14.9) | 405 (85.1) | Ref | |  |
| Diarrhea |  |  |  |  | |  |
| Yes | 143 | 18 (12.6) | 125 (87.4) | 0.114 (0.39 – 1.11) | | 0.11 |
| No | 363 | 68 (18.7) | 295 (81.3) | Ref | |  |
| Fever |  |  |  |  | |  |
| Yes | 369 | 60 (16.3) | 309 (83.7) | 0.86 (0.54 – 1.36) | | 0.51 |
| No | 137 | 26 (19.0) | 111 (81.0) | Ref | |  |
| Vomiting | | | | | | |
| Yes | 138 | 24 (17.4) | 114 (82.6) | 1.02 (0.63 – 1.63) | | 0.94 |
| No | 368 | 62 (16.8) | 306 (83.2) | Ref | |  |
| Signs on Physical Examination |  |  |  |  | |  |
| Oxygen saturation, percentage, mean (SD) | 506 | 93.2 (7.7) | 96.2 (3.9) | 0.93 (0.91 – 0.95) | | <0.001^¶^ |
| GCS (ordinal) | | | | | | |
| < 13 | 16 | 11 (68.8) | 5 (31.2) | 0.66 (0.60 – 0.73) | | <0.001^¶^ |
| 13-14 | 11 | 6 (54.5) | 5 (45.5) |  |  |  |
| 15 | 479 | 69 (14.4) | 410 (85.6) |  |  |  |
| Bilateral lower extremity edema | | | | | | |
| Yes | 67 | 20 (29.9) | 47 (70.1) | 2.31 (1.40 – 3.81) | | 0.001^¶^ |
| No | 439 | 66 (15.0) | 373 (85.0) | Ref | |  |
| Respiratory Rate, breaths per minute, mean (SD) | | | | | | |
| 2 – 5 years | 362 | 41.2 (16.9) | 33.0 (11.5) | 1.04 (1.02 – 1.06) | | <0.001^¶^ |
| 6 – 12 years | 144 | 32.1 (9.4) | 27.5 (11.8) | 1.02 (1.00 – 1.04) | | 0.03^¶^ |
| Diastolic blood pressure, mm Hg, mean (SD) | | | | | | |
| 2 – 5 years | 362 | 59.2 (10.7) | 60.0 (9.2) | 0.99 (0.96 – 1.02) | 0.52 | |
| 6 – 12 years | 144 | 63.2 (13.4) | 68.1 (9.0) | 0.94 (0.91 – 0.98) | 0.005^¶^ | |
| Heart rate, beats per minute, mean (SD) | |  |  |  |  | |
| 2 – 5 years | 362 | 121.1 (22.4) | 117 (22.0) | 1.00 (0.99 – 1.02) | 0.12 | |
| 6 – 12 years | 144 | 111.9 (20.1) | 106.4 (20.4) | 1.01 (0.99 – 1.02) | 0.12 | |
| Systolic blood pressure, mm Hg, mean (SD) | |  |  |  |  | |
| 2 – 5 years | 362 | 91.6 (14.8) | 90.2 (12.5) | 1.00 (0.98 – 1.02) | 0.52 | |
| 6 – 12 years | 144 | 100.4 (17.8) | 103.7 (12.4) | 0.97 (0.95 – 1.00) | 0.13 | |
| Nutritional status |  |  |  |  |  | |
| Severe Malnutrition ∆ | 71 | 15 (21.1) | 56 (78.9) | 1.49 (0.83 – 2.70) | 0.17 | |
| Moderate Malnutrition + | 76 | 13 (17.1) | 63 (82.9) | 1.07 (0.57 – 1.98) | 0.83 | |
| Mild Malnutrition * | 95 | 15 (15.8) | 80 (84.2) | 0.99 (0.55 – 1.77) | 0.96 | |
| Normal | 264 | 43 (16.3) | 221 (83.7) | Ref |  | |
| Temperature, Celsius, mean (SD) | 506 | 37.3 (1.2) | 37.2 (0.97) | 1.05 (0.84 – 1.30) | 0.68 | |
| Laboratory Investigation on Hospitalization | |  |  |  |  | |
| Hemoglobin level, g/dL, mean (SD) | 505 | 6.5 (2.8) | 8.1 (2.6) | 0.82 (0.75 – 0.88) | <0.001^¶^ | |
| Proteinuria by urinalysis (binary) | | | | | | |
| Positive | 97 | 28 (28.9) | 69 (71.1) | 2.38 (1.51 – 3.74) | <0.001^¶^ | |
| Negative | 409 | 58 (14.2) | 351 (85.8) | Ref |  | |
| Hematuria by urinalysis (binary) | | | | | | |
| Positive | 20 | 8 (40.0) | 12 (60.0) | 2.81 (1.35 – 5.81) | 0.005^¶^ | |
| Negative | 486 | 78 (16.0) | 408 (84.0) | Ref |  | |
| eGFR < 60 ml/min/1.73m^2^ (binary) | | | | | | |
| Yes | 106 | 27 (25.5) | 79 (74.5) | 1.91 (1.21 – 3.02) | 0.005^¶^ | |
| No | 400 | 59 (14.8) | 341 (85.2) | Ref |  | |
| eGFR, ml/min/1.73m^2^, mean (SD) | 506 | 106.9 (68.8) | 114.8 (57.8) | 0.99 (0.99 – 1.00) | 0.19 | |
|  | | | | | | |
| Random blood glucose, mg/dL, mean (SD) | 506 | 101 (32) | 108 (95) | 0.98 (0.90 – 1.05) | 0.53 | |
| Diagnosis Category | | | | |  | |
| Cancer | 20 | 12 (60.0) | 8 (40.0) | 11.79 (4.95-28.03) | <0.001^¶^ | |
| Heart disease | 23 | 10 (43.5) | 13 (56.5) | 7.11 (2.89 – 17.51) | <0.001^¶^ | |
| Sickle cell disease | 61 | 14 (23.0) | 47 (77.0) | 3.32 (1.44 – 7.68) | 0.005^¶^ | |
| Neurologic diseases | 33 | 8 (24.2) | 25 (75.8) | 3.51 (1.35 – 9.11) | 0.01^¶^ | |
| Septic shock | 14 | 4 (28.6) | 10 (71.4) | 4.64 (1.42 – 15.08) | 0.01^¶^ | |
| Severe malnutrition | 35 | 7 (20.0) | 28 (80.0) | 3.19 (1.18 – 8.57) | 0.02^¶^ | |
| Anemia | 49 | 7 (14.3) | 42 (85.7) | 2.03 (0.75 – 5.46) | 0.15 | |
| Diarrheal diseases | 60 | 8 (13.3) | 52 (86.7) | 1.94 (0.75 – 5.04) | 0.17 | |
| Other | 50 | 7 (14.0) | 43 (86.0) | 1.96 (0.73 – 5.27) | 0.18 | |
| Urinary tract infections | 36 | 0 (0) | 36 (100) | NA | NA | |
| Respiratory infections & Malaria | 124 | 9 (7.3) | 115 (92.7) | Ref |  | |

Data are presented as number (percentage) of study participants unless otherwise indicated.

Abbreviations: NA, not applicable

* Weight-for-Height Z score < -1 and ≥ -2 SD

+ Weight-for-Height Z score < -2 and ≥ -3 SD

∆ Weight-for-Height Z score < -3 SD

¶ P value significant (<0.05)
