# Supplementary material for: Post-hospital mortality in children aged 2-12 years in Tanzania: A prospective cohort study
Source: PLoS One. 2018 Aug 14;13(8):e0202334. doi: 10.1371/journal.pone.0202334 (PMC6091952; doi:10.1371/journal.pone.0202334)
Supplement: S2 Table — (DOCX) [file pone.0202334.s003.docx]

**S2 Table. Univariate Cox regression analysis for factors associated with post-hospital mortality.**

| **Variable** | **Total**  **N=467** | **Dead**  **N=47** | **Alive**  **N=420** | **Hazard Ratio**  **(95% CI)** | **p-value** |
| --- | --- | --- | --- | --- | --- |
| Demographic Characteristics |  |  |  |  |  |
| Age, months, mean (SD) | 467 | 69.2 (14.8) | 52.6 (11.2) | 1.01 (1.00 - 1.02) | 0.001^¶^ |
| Categorical Age | | | | | |
| Under 5 years | 306 | 21 (6.8) | 285 (93.1) | Ref |  |
| 5 – 12 years | 161 | 26 (16.1) | 135 (83.9) | 2.44 (1.37 – 4.34) | 0.002^¶^ |
| Lake or pond as water source | | | | | |
| Yes | 180 | 22 (12.2) | 158 (87.8) | 1.53 (0.86 – 2.72) | 0.14 |
| No | 287 | 25 (8.7) | 262 (91.2) | Ref |  |
| Sex | | | | | |
| Male | 272 | 26 (9.6) | 246 (90.4) | 0.91 (0.51 - 1.61) | 0.74 |
| Female | 195 | 21 (10.7) | 174 (89.2) | Ref |  |
| Pit latrine at home |  |  |  |  |  |
| Yes | 276 | 31 (11.2) | 245 (88.8) | 1.48 (0.81 – 2.71) | 0.19 |
| No | 191 | 16 (8.4) | 175 (91.6) | Ref |  |
| HIV Status |  |  |  |  |  |
| Positive | 25 | 1 (4.0) | 24 (96.0) | 0.43 (0.59 – 3.15) | 0.41 |
| Negative | 442 | 46 (10.4) | 396 (89.6) | Ref |  |
| Symptoms Reported on Hospitalization | |  |  |  |  |
| Diarrhea |  |  |  |  |  |
| Yes | 131 | 6 (4.6) | 125 (95.4) | 0.35 (0.15 – 0.83) | 0.018^¶^ |
| No | 336 | 41 (12.2) | 295 (87.8) | Ref |  |
| Fever | | | | | |
| Yes | 339 | 30 (8.8) | 309 (91.2) | 0.65 (0.36 – 1.19) | 0.16 |
| No | 128 | 17 (13.3) | 111 (86.7) | Ref |  |
| Decreased urine output | | | | | |
| Yes | 18 | 3 (16.7) | 15 (83.3) | 1.90 (0.59 – 6.13) | 0.28 |
| No | 449 | 44 (9.8) | 405 (90.2) | Ref |  |
| Vomiting |  |  |  |  |  |
| Yes | 129 | 15 (11.6) | 114 (88.4) | 1.22 (0.66 – 2.26) | 0.51 |
| No | 338 | 32 (9.4) | 306 (90.5) | Ref |  |
| Signs on Physical Examination |  |  |  |  |  |
| Oxygen saturation, percentage, mean (SD) | 467 | 94.6 (5.2) | 96.2 (3.9) | 0.94 (0.90 – 0.99) | 0.016^¶^ |
| Diastolic blood pressure, mm Hg, mean (SD) | | | | | |
| 2 – 5 years | 334 | 57.8 (7.1) | 60.0 (9.2) | 0.97 (0.93 – 1.01) | 0.21 |
| 6 – 12 years | 133 | 63.8 (8.8) | 68.1 (9.0) | 0.94 (0.90 – 0.99) | 0.02^¶^ |
| Systolic blood pressure, mm Hg, mean (SD) | | | | | |
| 2 – 5 years | 334 | 91.6 (13.3) | 90.2 (12.5) | 1.00 (0.97 – 1.03) | 0.65 |
| 6 – 12 years | 133 | 99.4 (7.1) | 103.7 (12.4) | 0.96 (0.93 – 1.00) | 0.07 |
| Respiratory Rate, breaths per minute, mean (SD) | | | | | |
| 2 – 5 years | 334 | 36.7 (17.4) | 33.0 (11.5) | 1.02 (0.99 – 1.05) | 0.12 |
| 6 – 12 years | 133 | 31.1 (9.8) | 27.5 (11.8) | 1.02 (0.99 – 1.05) | 0.13 |
| Temperature, Celsius, mean (SD) | 467 | 37.1 (0.95) | 37.2 (0.97) | 0.83 (0.60 – 1.14) | 0.25 |
| Bilateral lower extremity edema | | | | | |
| Yes | 53 | 6 (11.3) | 47 (88.7) | 1.21 (0.51 – 2.85) | 0.44 |
| No | 414 | 41 (9.9) | 373 (90.1) | Ref |  |
| Nutritional status | | | | | |
| Normal | 245 | 24 (9.7) | 221 (90.2) | Ref |  |
| Mild Malnutrition * | 91 | 11 (12.1) | 80 (87.9) | 1.32 (0.64 – 2.70) | 0.44 |
| Moderate Malnutrition + | 70 | 7 (10.0) | 63 (90.0) | 1.02 (0.44 – 2.37) | 0.95 |
| Severe Malnutrition ∆ | 61 | 5 (8.2) | 56 (91.8) | 0.98 (0.37 – 2.56) | 0.96 |
| Heart rate, beats per minute, mean (SD) | |  |  |  |  |
| 2 – 5 years | 334 | 116 (19.2) | 117 (22.0) | 1.00 (0.98 – 1.01) | 0.88 |
| 6 – 12 years | 133 | 108.3 (14.7) | 106.4 (20.4) | 1.00 (0.98 – 1.02) | 0.63 |
| GCS (ordinal) |  |  |  |  |  |
| < 13 | 7 | 2 (28.6) | 5 (71.4) | 0.75 (0.54 – 1.04) | 0.94 |
| 13-14 | 7 | 2 (28.6) | 5 (71.4) |  |  |
| 15 | 453 | 43 (9.5) | 410 (90.5) |  |  |
| Laboratory Investigation on Hospitalization | |  |  |  |  |
| Hemoglobin level, g/dL, mean (SD) | 467 | 6.3 (2.8) | 8.1 (2.6) | 0.78 (0.70 – 0.87) | <0.001^¶^ |
| Proteinuria by urinalysis (binary) | | | | | |
| Positive | 82 | 13 (15.9) | 69 (84.1) | 2.04 (1.08 – 3.88) | 0.028 |
| Negative | 385 | 69 (17.9) | 351 (91.1) | Ref |  |
| Hematuria by urinalysis (binary) | | | | | |
| Positive | 16 | 4 (25.0) | 12 (75.0) | 2.68 (0.96 – 7.48) | 0.059 |
| Negative | 451 | 43 (9.5) | 408 (90.5) | Ref |  |
| eGFR, ml/min/1.73m^2^, mean (SD) | 467 | 127.7 (68.3) | 114.8 (57.8) | 1.00 (0.99 – 1.00) | 0.15 |
| eGFR < 60 ml/min/1.73m^2^ (binary) | | | | | |
| Yes | 87 | 8 (9.1) | 79 (90.8) | 0.87 (0.41 – 1.88) | 0.740 |
| No | 380 | 39 (10.3) | 341 (89.7) | Ref |  |
| Random blood glucose, mg/dL, mean (SD) | 467 | 104 (30) | 108 (95) | 0.98 (0.91 – 1.06) | 0.77 |
| Diagnosis Category | | | | |  |
| Cancer | 13 | 5 (38.5) | 8 (61.5) | 7.85 (2.49 – 24.76) | <0.001^¶^ |
| Heart disease | 21 | 8 (38.1) | 13 (61.9) | 8.11 (2.94 – 22.37) | <0.001^¶^ |
| Neurologic diseases | 31 | 6 (19.4) | 25 (80.6) | 3.46 (1.16 – 10.2) | 0.02^¶^ |
| Sickle cell disease | 57 | 10 (17.5) | 47 (82.4) | 3.11 (1.18 – 8.17) | 0.02^¶^ |
| Anemia | 46 | 4 (8.7) | 42 (91.3) | 1.51 (0.44 – 5.16) | 0.50 |
| Diarrheal diseases | 54 | 2 (3.7) | 52 (96.3) | 0.63 (0.13 – 3.05) | 0.57 |
| Severe malnutrition | 30 | 2 (6.6) | 28 (93.3) | 1.25 (0.26 – 6.05) | 0.77 |
| Other | 46 | 3 (6.5) | 43 (93.4) | 1.08 (0.27 – 4.18) | 0.90 |
| Septic shock | 10 | 0 (0) | 10 (100) | NA | NA |
| Urinary tract infections | 36 | 0 (0) | 36 (100) | NA | NA |
| Respiratory infections & Malaria | 122 | 7 (5.7) | 115 (94.3) | Ref |  |

Data are presented as number (percentage) of study participants unless otherwise indicated.

Abbreviations: NA, not applicable

* Weight-for-Height Z score < -1 and ≥ -2 SD

+ Weight-for-Height Z score < -2 and ≥ -3 SD

∆ Weight-for-Height Z score < -3 SD

¶ P value significant (<0.05) for comparison of dead vs. alive
