# Supplementary material for: Post-hospital mortality in children aged 2-12 years in Tanzania: A prospective cohort study
Source: PLoS One. 2018 Aug 14;13(8):e0202334. doi: 10.1371/journal.pone.0202334 (PMC6091952; doi:10.1371/journal.pone.0202334)
Supplement: S3 Table — (DOCX) [file pone.0202334.s004.docx]

**S3 Table. Multivariate Cox regression analysis for factors associated with post-hospital mortality.**

| **Variable** | **Hazard Ratio**  **(95% CI)** | **p-value** |
| --- | --- | --- |
| Hemoglobin level | 0.79 (0.70 – 0.88) | <0.001^¶^ |
| Age | 1.01 (1.00 – 1.01) | 0.006^¶^ |
| Oxygen saturation | 0.93 (0.89 – 0.98) | 0.008^¶^ |
| Proteinuria by urinalysis | 2.13 (1.12 – 4.05) | 0.021^¶^ |
| Diarrhea | 0.49 (0.20 – 1.17) | 0.11 |

¶ P value significant (<0.05)
